# Place-based, intersectional variation in caregiving patterns and health outcomes among informal caregivers in the United States

**DOI:** 10.3389/fpubh.2024.1423457

**Published:** 2024-08-19

**Authors:** Steven A. Cohen, Caitlin C. Nash, Mary L. Greaney

**Affiliations:** Department of Public Health, University of Rhode Island, Kingston, RI, United States

**Keywords:** rural health, informal caregiver, disparities (health racial), caregiver health outcomes, effect modification

## Abstract

**Introduction:**

Informal caregiving is a critical component of the healthcare system despite numerous impacts on informal caregivers’ health and well-being. Racial and gender disparities in caregiving duties and health outcomes are well documented. Place-based factors, such as neighborhood conditions and rural–urban status, are increasingly being recognized as promoting and moderating health disparities. However, the potential for place-based factors to interact with racial and gender disparities as they relate to caregiving attributes jointly and differentially is not well established. Therefore, the primary objective of this study was to jointly assess the variability in caregiver health and aspects of the caregiving experience by race/ethnicity, sex, and rural–urban status.

**Methods:**

The study is a secondary analysis of data from the 2021 and 2022 Behavioral Risk Factor Surveillance System (BRFSS) from the Centers for Disease Control and Prevention. Multivariable logistic regression or Poisson regression models assessed differences in caregiver attributes and health measures by demographic group categorized by race/ethnicity, sex, and rural–urban status.

**Results:**

Respondents from rural counties were significantly more likely to report poor or fair health (23.2% vs. 18.5%), have obesity (41.5% vs. 37.1%), and have a higher average number of comorbidities than urban caregivers. Overall, rural Black male caregivers were 43% more likely to report poor or fair health than White male caregivers (OR 1.43, 95% CI 1.21, 1.69). Urban female caregivers across all racial groups had a significantly higher likelihood of providing care to someone with Alzheimer’s disease than rural White males (*p* < 0.001). Additionally, there were nuanced patterns of caregiving attributes across race/ethnicity*sex*rural–urban status subgroups, particularly concerning caregiving intensity and length of caregiving.

**Discussion:**

Study findings emphasize the need to develop and implement tailored approaches to mitigate caregiver burden and address the nuanced needs of a diverse population of caregivers.

## Introduction

Informal caregiving is a critical component of the United States (US) healthcare system, saving the national economy over $500 billion annually that may otherwise be spent on costly long-term care expenses for older adults with disabilities, cognitive decline, and other chronic conditions (1). Nearly one in six Americans is an informal caregiver (2). Protecting the health and well-being of the 40 million informal caregivers across the US is essential for protecting the care recipients’ health and sustaining the healthcare system and the national economy. However, informal caregiving impacts nearly every aspect of the caregivers’ life. The extent to which caregiving adversely affects informal caregivers’ emotional, social, financial, physical, and spiritual functioning—caregiver burden—is often overlooked. Informal caregivers for older adults face varying degrees of caregiver burden (3–5). The level and type of care provided (6), social support (7), socioeconomic (8), demographic (9), and environmental factors (10) at the individual, community, and organizational levels contribute to and moderate the degree and type of caregiver burden experienced (11).

The type and amount of caregiving provided and the impacts on caregiver health vary across demographic groups (12, 13). There are well-documented disparities in caregiver burden and other consequences of caregiving, including strain and health-related quality of life by sex or gender (14, 15). Furthermore, there are differences in the intensity of caregiving, with female caregivers providing substantially more intensive care than their male counterparts (16, 17). Gender disparities in caregiving intensity only partially explain differences in caregiver burden (18), but more research is needed to understand these complex associations more fully.

Racial and ethnic differences in caregiving intensity and caregiver burden are well documented (19, 20). Black informal caregivers provide substantially higher levels of care than their White counterparts (21). However, Black caregivers do not proportionately report higher levels of caregiver burden and health impacts (22). A study of male informal caregivers determined that the correlates of caregiver burden differed by race/ethnicity, where Black caregivers of the sandwich generation—those with at least one child under age 18 living at home—experienced greater burden compared to those without a child in the home. In comparison, physical pain and fatigue experienced by the caregiver were predictive of higher caregiver burden among Hispanic caregivers (23).

Recent research in informal caregiving has expanded on the study of racial/ethnic and gender differences in caregiver burden and aspects of caregiving by using an intersectional framework to examine the potential for these two factors to impact caregiving jointly and differentially (24). Broadly, intersectionality is a theoretical framework that conceives multiple social categories, such as race/ethnicity and gender, interact to reflect multiple linked systems of privilege and oppression, such as racism and sexism (25). The intersectional framework posits that factors such as race/ethnicity, gender, socioeconomic status (SES), and other social identities create systems of disparities above and beyond the effect of any single factor (26–28). A 2019 study found that Black caregivers spent an average of 28.5 more hours/month caregiving than White caregivers and that Black female caregivers provided significantly higher intensity care than White females and White and Black males (9). However, some research has determined that White female informal caregivers report greater emotional strain than female caregivers of color (29). It has been postulated that these differences in caregiver strain may be partly attributable to differences in resilience across racial and gender groups (30), but further study is needed to clarify these relationships.

Furthermore, there is increasing recognition that place-based or geographic factors contribute to differences in informal caregiving and caregiver health. That is, there are notable differences by rural–urban status. Informal caregiving in rural areas presents unique challenges to the caregiver, including increased distance to, or lack of, caregiving resources and supports (31); increased social isolation (32), decreased access to high-quality health care (33), and farther travel to the care recipient’s residence if the care recipient does not reside with the caregiver (34). As a result, rural caregivers face substantial barriers to acquiring and providing caregiving-related support and experience greater difficulty caring for their own health. They are also less likely to have health insurance than urban informal caregivers (35).

To date, no comprehensive assessment of rural caregivers with respect to general health, comorbidities, and aspects of the caregiving experience has been conducted, nor has there been a comparison of existing racial/ethnic and gender-or sex-based disparities across the rural–urban spectrum among caregivers concerning caregiver health and caregiving attributes (36). A vast body of existing research has examined race/ethnicity, gender, and rural–urban status separately. Several studies have investigated the joint effects of two intersecting factors (e.g., race/ethnicity and gender). For example, one study assessed the joint influence of race and gender on creating disparities in caregiving and caregiver health with a sample of rural caregivers (12), but no rural–urban comparisons were conducted. A seminal review of rural health emphasized the need to delve deeper into rural–urban disparities (37). The authors emphasize that to fully understand and improve population health in rural areas, research must consider other structural and intersectional determinants of health within rural communities and compare rural to urban areas. Other studies underscore the need to research the intersections of economic wellbeing and family structure with rural health and aging and how social and physical isolation inherent to rural areas has differential impacts for older adults and their caregivers (38). To date, no studies have assessed the potential for associations between caregiver demographics and caregiving experiences and health outcomes to vary by geography. There is, therefore, a compelling need to identify, understand, and address the potential for these intersecting demographic and place-based factors that result in complex disparities in informal caregiver health, caregiver burden, and overall caregiving experience (26). This study applies and extends the theoretical framework of intersectionality to include not only individual attributes (e.g., race/ethnicity and sex), but also place of residence (rural vs. urban). The primary objective of this exploratory study was to assess potential variability with respect to caregiver health and aspects of the caregiving experience jointly by race/ethnicity, sex, and rural–urban status.

## Methods

### Data source and analytic sample

This was a secondary analysis of data from the 2021 and 2022 Behavioral Risk Factor Surveillance System (BRFSS), the largest system of health-related telephone surveys administered by the Centers for Disease Control and Prevention (CDC). Respondents were selected and then interviewed through landlines or cell phones. The BRFSS collects data annually from US residents aged 18+ in all 50 states and Puerto Rico regarding their demographics, self-reported health-related risk behaviors, height, weight, chronic health conditions, and use of preventive services. The data collected are widely used for policy and program planning, largely at the state level (39). Each year, between 400,000 and 500,000 interviews are conducted, with a total sample of 438,693 respondents in 2021 and 441,132 in 2022. Response rates for 2021 and 2022 were 44.0 and 45.0%, respectively (40). Data from 2021 and 2022 were combined for statistical analysis for this study.

The BRFSS Caregiver Module is an optional set of nine questions concerning whether the respondent is an informal caregiver. Individual states decide whether to include this module in their annual questionnaire. In 2021, the Caregiver Module was administered in 39 states; in 2022, it was administered in 14 states. Collectively, between the 2 years, the module was administered in 47 states—all except Florida, Montana, and Tennessee. Persons identifying as caregivers complete several questions assessing caregiving, including the type of caregiving, hours per week spent caregiving, and duration of caregiving. The analytic sample for this study was restricted to respondents in either data set who responded “yes” to whether they were informal caregivers. The resultant sample size was n = 74,822 respondents.

### Outcome measures

#### Health and health-related quality of life

Four primary outcome variables on health and health-related quality of life were obtained. Respondents were asked to rate their general health as “excellent,” “very good,” “good,” “fair,” or “poor.” Responses were dichotomized into two categories (fair or poor vs. excellent, very good, and good) (41). Respondents’ self-reported height and weight were used to calculate BMI, which was used to ascertain obesity status. Respondents whose BMI was 30 kg/m^2^ or above were classified as having obesity, while those with a BMI below 30 kg/m^2^ were classified as not having obesity. The third variable was whether the respondent reported having depressive disorders (yes vs. no). Lastly, a variable containing the sum of major reported comorbidities was calculated from the following measures: diabetes, cancer, hypercholesterolemia, heart disease, myocardial infarction, stroke, hypertension, asthma, chronic obstructive pulmonary disorder, and kidney disease. Comorbidity scores could range from 0 to 10.

### Aspects of caregiving

Five aspects of the caregiving experience were examined. The first, a measure of the length of time providing care, was dichotomized into 6 months or more vs. less than 6 months. The second, a measure of hours of caregiving per week, was dichotomized into at least 20 h vs. less than 20 h, in accordance with how intensity caregiving is defined in a recent CDC report (42). The third measure asked whether the care recipient had Alzheimer’s disease (yes vs. no). The last two measures addressed the type of caregiving: whether or not the caregiver provides personal care in the form of activities of daily living (ADLs) to help with tasks such as toileting, eating, bathing, and dressing, and the other about household caregiving, instrumental activities of daily living (IADLs), such as paying bills, medication management, and transportation. Both were dichotomous responses (no ADLs or IADLs vs. at least one).

### Exposure measures

Respondents were asked, “Which one of these groups would you say best represents your race?” Eight response options were available: White, Black, Asian, Native Hawaiian or Other Pacific Islander, American Indian or Alaska Native, other, do not know, and multiracial. They were also asked if they were of Hispanic ethnicity (yes vs. no). Responses for these two questions were combined into a categorical variable consisting of four categories: White, Black, Hispanic, and Other. The Other category was necessary due to the small sample sizes among respondents identifying as Asian, Native Hawaiian or Other Pacific Islander, American Indian or Alaska Native, other, and multiracial. The other major exposure measure was sex. Each respondent was asked about their sex assigned at birth. This dichotomous variable (female vs. male) was used in the analysis. Lastly, rural–urban status was based on each respondent’s county of residence from the BRFSS data set and was a dichotomous variable (rural vs. urban).

### Covariates

Other covariates used were the 5-year age category (except for the first category, which was 18–24), marital status (currently married vs. not currently married), education (less than bachelor’s degree vs. bachelor’s degree or higher), current employment status (currently employed for pay, not employed, retired, or student), and annual household income category (<$50,000, $50,000-99,999, $100,000+, and missing/unknown).

### Data analysis

Univariate descriptive statistics were obtained for all study variables—outcomes, exposures, and covariates. Frequencies (N and %) were assessed for all categorical variables and means, and standard deviations were obtained for all continuous and count variables. State-level geographic distributions of each of the nine main outcome measures—health, health-related quality of life, and aspects of caregiving—were assessed through mapping. Chi squared statistics were used to assess bivariate associations between each categorical variable and rural–urban status, and Wilcoxon rank sum tests were used to assess the bivariate association between rural–urban status and the number of comorbidities, a count variable.

Weighted multiple binary logistic regression analyses were used to evaluate the associations between the nine outcome variables and each sex*race/ethnicity*rural–urban status population subgroup, accounting for covariates using the sample weights provided in the BRFSS datasets. Respondents were cross-classified by sex, race/ethnicity, and rural–urban status into one of 16 race/ethnicity*sex*rural–urban indicator variables, as the purpose of this exploratory analysis was to evaluate these three exposures simultaneously. The subgroup of urban White males served as the reference group in all models, and the remaining 15 subgroups were compared to that group. Covariates included in the models were current marital status (reference group = not married), education (reference = less than bachelor’s degree), employment (reference = not employed), age in 5-year intervals, annual income (reference = < $50,000), and an indicator variable of which year the observation was derived (2021 or 2022). Model fit was assessed using Akaike’s Information Criterion (AIC) and the Cox & Snell and Nagelkerke r-squared values. Missing data was assumed to be at random. Statistical significance was set to *p* < 0.05. SAS version 9.4 (Cary, NC) and IBM SPSS version 29 (Armonk, NY) were used for data management and analysis.

## Results

Descriptive statistics by rural–urban status for major exposures, covariates, and outcome measures are provided in Table 1. The final analytic sample size was *n* = 74,822, of which 85.9% were from urban counties and 14.1% were from rural counties. Compared to those from urban counties, respondents from rural counties were more likely to be aged 65 and over (38.1% vs. 31.4%), White (84.1% vs. 72.8%), and currently married (63.3% vs. 58.5%) (*p* < 0.001 for all). Respondents from rural counties were less likely to hold at least a bachelor’s degree (30.0% vs. 43.5%), be currently employed (46.1% vs. 51.6%), and have an annual household income of $100,000 or more (*p* < 0.001 for all). Respondents from rural counties were more likely to report poor or fair general health (23.2% vs. 18.5%), have obesity (41.5% vs. 37.5%), and have a greater average number of comorbidities (0.69 vs. 0.60) than their urban counterparts. Although rural respondents were significantly less likely to provide personal (48.6% vs. 49.0%) or household (78.3% vs. 79.5%) care than their urban counterparts, they were more likely to have been caregiving for at least 6 months (72.7% vs. 72.1%) and perform at least 20 h per week caregiving (32.0% vs. 31.0%). Urban caregivers were 8.9% more likely to care for someone with Alzheimer’s disease than rural caregivers (*p* < 0.001).

**Table 1:** Frequencies of major exposure and outcome variables by rural–urban status.

		Urban	Rural	*p*-value
	*N* (%)	64,304 (85.9)	10,518 (14.1)	
		Weighted %	Weighted %	
Age group	18–39	18.7	15.1	<0.001
	40–64	49.9	46.8	
	65+	31.4	38.1	
Sex	Female	60.7	60.6	0.136
	Male	39.3	39.4	
Race/ethnicity	White	72.8	84.1	<0.001
	Black	10.4	7.7	
	Asian	2.3	0.2	
	Hispanic	9.5	3.2	
	Other	5.0	4.8	
Education	Bachelor’s or higher	43.5	30.0	<0.001
	Less than bachelor’s	56.5	70.0	
Employment	Currently employed	51.6	46.1	<0.001
	Not employed	18.5	19.6	
	Retired	28.1	33.1	
	Student	1.9	1.3	
Annual income ($)	<50 k	34.5	44.2	<0.001
	50–99.9 k	26.3	25.2	
	100 k+	22.6	12.3	
Currently married	Yes	58.5	63.3	<0.001
	No	41.5	36.7	
General health	Poor or fair	18.5	23.2	<0.001
	Good, very good, or excellent	81.5	76.8	
Has obesity	Yes	37.4	41.5	< 0.001
	No	62.6	58.5	
Has depressive disorders	Yes	25.6	25.4	0.055
	No	74.4	74.6	
Average number of comorbidities	Mean (SD)	0.60 (0.93)	0.69 (1.00)	<0.001
Length of providing care	6 months or more	72.1	72.7	<0.001
	Less than 6 months	27.9	27.3	
Hours of caregiving per week	20 h or more	31.0	32.0	<0.001
	Less than 20 h	69.0	68.0	
Care recipient has Alzheimer’s disease	Yes	14.9	13.7	<0.001
	No	85.1	86.3	
Provides personal (ADL) care	Yes	49.0	48.6	<0.001
	No	51.0	51.4	
Provides household (IADL) care	Yes	79.5	78.3	<0.001
	No	20.5	21.7	

**Figure 1:**
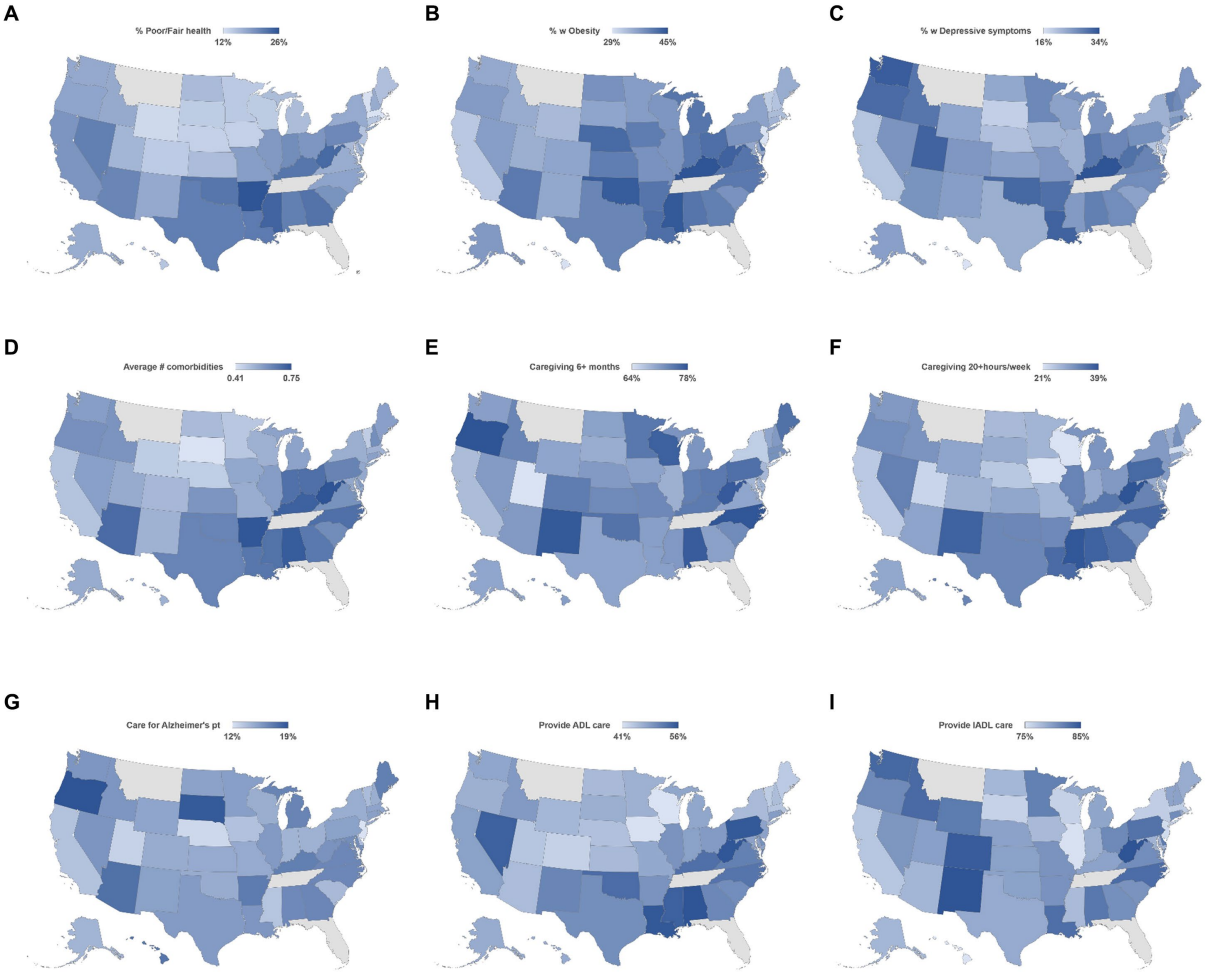
Geographic distribution of health issues **(A–D)** and caregiving attributes **(E–I)** by state. Percent with poor or fair health **(A)**, having obesity **(B)**, with depressive symptoms **(C)**, and average number of comorbidities **(D)**; Percent of caregivers providing caregiving for 6+ months **(E)**, providing care for 20+ hours per week **(F)**, providing care to Alzheimer’s patient **(G)** providing ADL care **(H)**, and providing IADL care **(I)**.

The geographic distributions of each of the nine main outcome variables are shown in Figure 1. The percentage of informal caregivers reporting poor or fair health (Panel A) and obesity (Panel B) trended highest in the Southern and lower Midwest states. The highest percentage of caregivers reporting depressive symptoms (Panel C) occurred in Kentucky (33.7%) and Washington (32.5%), with the lowest rates occurring in Hawaii (16.1%), South Dakota (18.6%), and New Jersey (19.2%). Caregivers from Southern states also had some of the highest average number of comorbidities (Panel D), with West Virginia (0.75 average comorbidities), Arkansas (0.75), and Kentucky (0.72) with the highest values. Although there was no clear pattern in the spatial distributions of those providing care for at least 6 months (Panel E), caregivers from the Southern states were more likely to provide at least 20 h per week of caregiving than those from other areas (Panel F). The percentage of caregivers providing care for a patient with Alzheimer’s disease was highest in Oregon (18.8%) and South Dakota (18.5%) and lowest in New Jersey (11.6%) and Nebraska (12.2%) (Panel G). The percentage of caregivers providing personal (ADL-type) care was again highest in many Southern states, as well as Nevada and Pennsylvania (Panel H). Simultaneously, there was no discernible spatial pattern for those caregivers providing household care (Panel I).

**Figure 2:**
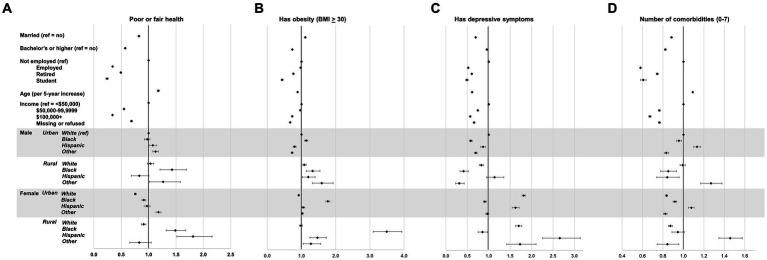
Weighted, adjusted odds ratios of health outcomes by race/ethnicity*sex*rural–urban status groupings and covariates. Percent with poor or fair health **(A)**, having obesity **(B)**, with depressive symptoms **(C)**, and average number of comorbidities (prevalence ratio) **(D)**.

Figure 2 shows adjusted odds ratios and 95% confidence intervals for the likelihood of reporting poor or fair general health (Panel A), having obesity (Panel B), and reporting depressive symptoms (Panel C), as well as incidence ratios for the number of comorbidities (Panel D). Rural Black male caregivers were significantly more likely to report poor or fair health than White male caregivers (OR 1.43, 95% CI 1.21, 1.69). However, there was no significant association for urban Black male or rural White male caregivers for this general health outcome. Among male caregivers, the likelihood of having obesity was significantly higher among urban Black males (OR 1.14, 95% CI 1.09, 1.19). Similar findings were observed for all rural population subgroups, including White people (OR 1.09, 95% CI 1.04, 1.14), Black people (OR 1.33, 95% CI 1.14, 1.54), Hispanic people (OR 1.20, 95% CI 1.03, 1.40), and people of other races and ethnicities (OR 1.59, 95% CI 1.32, 1.93), compared to White urban people. Similarly, many other population subgroups were significantly more likely to have obesity than urban White male caregivers, most notably urban female Black caregivers (OR 1.77, 95% CI 1.71, 1.83) and rural Black female caregivers (OR 3.49, 95% CI 3.10, 3.93). Compared to urban White male caregivers, Black (OR 0.58, 95% CI 0.55, 0.61), Hispanic (OR 0.87, 95% CI 0.81, 0.92), and Other (OR 0.70, 95% CI 0.67, 0.73) male caregivers in urban areas, along with rural White (OR 0.83, 95% CI 0.78, 0.88), Black (OR 0.41, 95% CI 0.33, 0.52), and Other (OR 0.31, 95% CI 0.22, 0.43) male caregivers in rural areas had significantly lower likelihood of having depressive symptoms. However, most female caregiver subgroups had a significantly higher likelihood of having depressive symptoms than urban White male caregivers, except Black female caregivers from urban (OR 0.91, 95% CI 0.87, 0.94) and rural areas (OR 0.85, 95% CI 0.75, 0.97).

**Figure 3:**
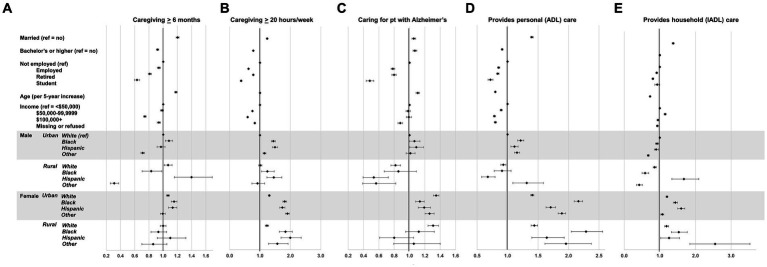
Weighted, adjusted odds ratios of caregiver attributes by race/ethnicity*sex*rural–urban status groupings and covariates. Percent of caregivers providing caregiving for 6+ months **(A)**, providing care for 20+ hours per week **(B)**, providing care to Alzheimer’s patient **(C)** providing ADL care **(D)**, and providing IADL care **(E)**.

Differences in attributes of caregiving by the examined factors are shown in Figure 3. Among urban male caregivers, Black caregivers were significantly more likely to have provided care for at least 6 months (OR 1.08, 95% CI 1.03, 1.13), at least 20 h of care per week (OR 1.44, 95% CI 1.37, 1,51), and personal (ADL) care (OR 1.21, 95% CI 1.16, 1.27), but significantly less likely to provide household (IADL) care (OR 0.92, 95% CI 0.88, 0.97) than White caregivers. Rural White male caregivers were more likely to have provided care for at least 6 months (OR 1.07, 95% CI 1.02, 1.13) but were less likely to provide ADL care (OR 0.93, 95% CI 0.89, 0.97) or ADL care (OR 0.85, 95% CI 0.81, 0.90) than urban White male caregivers. Among urban female caregivers, nearly all racial/ethnic subgroups were significantly more likely to have provided care for at least 6 months (except for those of the Other race/ethnicity category), provide at least 20 h per week of care, care for a patient with Alzheimer’s disease, and provide ADL and IADL care than urban White male caregivers. Similarly, among rural female caregivers, all racial/ethnic subgroups were more likely to provide at least 20 h of care per week and provide ADL care than urban White female caregivers.

## Discussion

This study identified substantial differences in the scope and intensity of caregiving, health, and health-related quality of life across demographic groups of informal caregivers. The identified associations were not uniform across the caregiving and health outcomes or race/ethnicity, sex, and rural–urban status. For example, the associations between race/ethnicity and caregiving hours varied notably by sex and rural–urban status, indicating the interdependence of demographic factors in predicting caregiver outcomes.

Another notable finding highlights the geographic distribution of these caregiving attributes and health outcomes among caregivers across the US. One example is in the prevalence of depressive symptoms identified among informal caregivers: caregivers in Kentucky are more than twice as likely to have depressive symptoms than caregivers living in Hawaii. Likewise, caregivers in many Southern states had higher intensity caregiving with respect to hours per week spent caregiving and provision of ADL care compared to caregivers in other regions. Collectively, the study findings emphasize that the population of 40+ million caregivers in the US is far from a monolith and that the experiences and consequences of informal caregiving vary widely by most of these measures. The potential mechanisms for this deserve further study. That said, variability in state-level policies may contribute to differences in caregiving experience and subsequent health impacts on informal caregivers (43). Cultural attitudes, family norms and expectations, and availability of support and formal care services may also explain some of the observed differences (44). In addition, lack of knowledge related to resources, as well as financial difficulties and poorer overall health both for the caregiver and care recipient may also play a role (45).

One important study finding was that, irrespective of rural–urban status, Black caregivers, particularly women, provide higher intensity caregiving than their White counterparts with respect to caregiving hours and providing ADL and IADL care, which is consistent with previous research (21, 46). It should be noted that the magnitude of these associations varied somewhat between urban and rural caregivers. A seminal paper by Dilworth-Anderson et al. (47) suggests that cultural differences, particularly regarding social roles, may explain the stark and consistent differences in caregiving roles and intensity by race and ethnicity, which persist across geographies. Utilization of paid or formal caregivers is more common among white person than other races (48), possibly due to financial and/or cultural factors (49). Interestingly, there was no clear and consistent pattern of higher prevalences of adverse health outcomes for those populations, supporting the hypothesis of higher resilience in those racial and ethnic groups (30). Social, religious, and cultural factors may help explain the relative resilience these caregivers have, such as familism and filial piety, which may offset the overall psychological toll of caregiving and allow for better coping (50, 51).

Also, urban female caregivers across all racial groups had a significantly higher likelihood of providing care to someone with Alzheimer’s disease. These findings were unexpected, given that the rate of Alzheimer’s disease and related dementias is 64% higher among Black older adults compared to White older adults (52). There are several possible explanations for these observations. One potential explanation is self-selection. Rural caregivers, regardless of race and ethnicity, have greater logistical (financial and healthcare-related) barriers than their urban counterparts (33) and face substantial barriers to support services (53, 54). Therefore, the rural setting may be less conducive for successful caregiving to Alzheimer’s patients, and caregivers may make the decision to move toward more urban or suburban regions to gain access to vital resources and services (45). Furthermore, from 1999 to 2018, mortality due to Alzheimer’s disease and related dementias increased nationwide, but this increase was more pronounced in rural areas than in urban areas (55). Other research suggests that underdiagnosis of Alzheimer’s disease in rural areas may also contribute to these disparities (56). More research is needed to assess what specific elements of rural vs. urban settings contribute to these disparities.

Another notable set of findings is the more nuanced patterns indicating differences in caregiving attributes simultaneously by race/ethnicity, sex, and rural–urban status. Such findings are evident in long-term (at least 6 months) caregiving. For this attribute, there was strong variation among the 16 race/ethnicity*sex*rural–urban subgroups. Male caregivers of other races/ethnicities in urban and rural settings were significantly less likely than White male urban caregivers to provide care for at least 6 months. However, urban Black male and female caregivers, rural Hispanic male caregivers, and urban Hispanic female caregivers were significantly more likely to have provided at least 6 months of care. Previous research supports these findings (57), underscoring the possibility that cultural factors (58), as well as financial constraints (59, 60) may account for such differences, by race/ethnicity, sex, and geography. Concurrently, there were no associations between the rural female caregiver subgroups for length of caregiving. Similar variability among caregiver subgroups, but slightly different patterns, were observed for personal and household care. Although the reasons for these patterns are unclear, these findings have particular significance in creating efforts to reduce caregiver burden and promote health equity. These findings suggest that any such efforts need to be uniquely tailored to the population subgroups most at risk and address their distinctive set of caregiving patterns that may promote caregiver stress and other negative impacts of caregiving.

There are several important limitations to consider when interpreting the study results. First, since the study used cross-sectional data, it is impossible to assess temporality or causation. Second, one of the three primary exposures was biological sex, not gender. Although the 2021 and 2022 BRFSS data sets do contain a variable on gender, it was contained in an optional module and, therefore, was only asked of approximately 61% of all BRFSS respondents. If gender is incorporated in the complete survey asked in all states in future BRFSS data sets, that variable could be used instead of biological sex in subsequent studies. A third limitation is the measure of rural–urban status. The dichotomous variable may mask more nuanced attributes in the rural–urban continuum (61) and may impact the observed associations between rural and urban caregivers (62). Furthermore, there is no universal measure of rural–urban status in the population health and gerontological literature (63). There is evidence that the current array of rural–urban status measures available will provide differing estimates of associations depending on which measure is used (64) and which of the many attributes of rural–urban status are emphasized in each measure (65, 66). Also, due to the limited questions on caregiving in the BRFSS module, subjective caregiver burden can not be assessed, which may complement the more objective measures used to provide a more thorough picture of the caregiving experience with respect to resources, cultural attitudes, and social support. Lastly, the present study was limited to the variables available in the BRFSS data and is based on self-report. Although several attributes of caregiving experiences, health, and health-related quality of life were assessed, it was not possible to examine other aspects of caregiving, such as caregiver burden, burnout, and socio-emotional strain, based on the use of these data.

The study has several notable strengths, as well. First, it is one of the first studies to incorporate intersections of multiple demographic factors, along with rural–urban status, a key place-based determinant of health, into evaluating their associations with aspects of caregiving and caregiver health using a large, nationally representative sample of informal caregivers. In addition, since the Caregiver Module was administered in 47 US states, the generalizability of the findings to US caregivers is robust. The states that are not represented—Florida, Montana, and Tennessee—are not centralized in one region. Future studies could examine data from previous BRFSS samples to determine if caregiving in those states varied substantially from the other 47 states. Although the analysis only addressed nine outcomes, four health and health-related quality of life measures, and five attributes of caregiving, it included many outcomes, many of which are policy actionable.

Study findings show substantial variability with respect to the caregiver’s race/ethnicity and sex, as well as where the caregiver lives with respect to many aspects of the caregiving experience and health conditions. Such results emphasize the need to address caregiver needs through effective policies, programs, and interventions on a highly granular level to reduce disparities and promote health equity. What may be effective in one population may not be effective in another. Further research can identify the specific, policy-actionable mechanisms that drive the observed differences in caregiving attributes and caregiver health and quality of life. Identifying and addressing these factors may have additional benefits not only to informal caregivers but also to the larger population who are subject to the same factors (e.g., race/ethnicity, sex, SES, place-based characteristics) that also promote other types of health disparities. As the demand for informal caregivers will continue to grow as the population continues to age, the need to develop and implement effective strategies to mitigate caregiver burden and address the nuanced needs of a diverse population of caregivers, with the ultimate goal of protecting and supporting this critical component of the healthcare system.

## Data Availability

The original contributions presented in the study are included in the article/supplementary material, further inquiries can be directed to the corresponding author/s.
